# Cloning and Analysis of a Large Plasmid pBMB165 from *Bacillus thuringiensis* Revealed a Novel Plasmid Organization

**DOI:** 10.1371/journal.pone.0081746

**Published:** 2013-12-02

**Authors:** Yueying Wang, Donghai Peng, Zhaoxia Dong, Lei Zhu, Suxia Guo, Ming Sun

**Affiliations:** State Key Laboratory of Agricultural Microbiology, College of Life Science and Technology, Huazhong Agricultural University, Wuhan, Hubei, People’s Republic of China; Universidad Nacional de La Plata., Argentina

## Abstract

In this study, we report a rapid cloning strategy for large native plasmids via a contig linkage map by BAC libraries. Using this method, we cloned a large plasmid pBMB165 from *Bacillus thuringiensis* serovar *tenebrionis* strain YBT-1765. Complete sequencing showed that pBMB165 is 77,627 bp long with a GC-content of 35.36%, and contains 103 open reading frames (ORFs). Sequence analysis and comparison reveals that pBMB165 represents a novel plasmid organization: it mainly consists of a pXO2-like replicon and mobile genetic elements (an inducible prophage BMBTP3 and a set of transposable elements). This is the first description of this plasmid organization pattern, which may result from recombination events among the plasmid replicon, prophage and transposable elements. This plasmid organization reveals that the prophage BMBTP3 may use the plasmid replicon to maintain its genetic stability. Our results provide a new approach to understanding co-evolution between bacterial plasmids and bacteriophage.

## Introduction

The *Bacillus cereus* *sensu lato* (*Bc*) group are rod-shaped gram-positive bacteria that are ubiquitous in the natural environment. The strict *Bc* group includes *Bacillus anthracis*, *Bacillus cereus*, and *Bacillus thuringiensis*. Since the *Bc* group bacteria are highly genetically homogeneous, the plasmids, especially large ones, usually carry pathogenicity-related and other functional genes, and play very important roles because of their different phenotypic properties [1].

As a mammalian pathogen, the ability of *B. anthracis* to cause anthrax originates from two large plasmids. The 182 kb plasmid pXO1 encodes the anthrax toxin genes, edema factor, lethal factor, and protective antigen, while the gene products from pXO2 (95 kb) synthesize an antiphagocytic poly-D-glutamic acid capsule [2]. *B. cereus* is well known as an important food contaminant, which can induce an emetic or a diarrheal type of food-associated illness [3]. It has been shown that the 24 kb cereulide synthetase gene cluster, which synthesizes the cereulide toxin that causes the emetic illness, is located on a large plasmid, pCER270 [4]. *B. thuringiensis* produces insecticidal crystals during its sporulation phase, and these crystal proteins are toxic to insect larvae, nematodes, mites, and protozoa [5]. Most of the reported crystal protein genes (*cry*) are located on large plasmids, except *cry55Aa1* and *cry6Aa2*, which are located on a 17.7 kb plasmid, pBMB0228 [6,7]. The gene clusters that synthesize Thuringiensin and Zwittermicin A are also located on large plasmids in *B. thuringiensis* [8-10]. Therefore, large plasmids are key components of the *Bc* group genome that usually enhance their hosts, pathology-associated bacteria, with the ability to acclimate and develop their environment [11]. 

With completed cloning and sequencing, researchers have found many more pathogenicity-related genes on the plasmid pBtoxis [12]. Whole sequence analysis has shown that several plasmids in different pathology-associated *Bc* group bacteria, with hosts ranging from humans to insects, have a high degree of similarity with the virulence plasmids pXO1 and pXO2 from *B. anthracis*. This reveals the widespread distribution of pXO1- and pXO2-like plasmids in the environment. pXO2 has an active evolutionary process, which may help in understanding the origin and evolution of the plasmids among *Bc* group bacteria [1,13,14]. These findings indicate that complete cloning and sequencing of large plasmids is especially important for studies on pathogenic bacteria.

Previously, we identified a 20 kb fragment harboring the replicon *ori165* of the plasmid pBMB165 from *B. thuringiensis* serovar tenebrionis YBT-1765. This is homologous to the pAMβ1 family replicons, especially the pXO2 replicon [15]. Differing from the typical pXO2-like plasmids pXO2, pAW63 and pBT9727, the 20 kb fragment of pBMB165 belongs entirely to a mobile genetic element. The density of transposon genes around the replicon indicates that pBMB165 may be a special pXO2-like plasmid. Here, we present a genomic BAC cloning strategy for complete cloning and sequencing of the large plasmid pBMB165. BAC clones provided an efficient way to completely clone, link and sequence the contigs. The complete sequence of pBMB165 showed a completely different organization to other plasmids. It consists of mobile genetic elements, including a 50 kb inducible prophage BMBTP3 and a 23 kb region rich in transposable elements, as opposed to the virulence and conjugation genes carried by other pXO2-like plasmids. This novel organization of pBMB165 provides a new outlook for understanding the origin and flexibility of bacterial plasmids and prophages during the process of evolution.

## Materials and Methods

### Bacterial artificial chromosome (BAC) library construction

The construction of the total plasmid and genomic BAC libraries of YBT-1765 were carried out as previously reported, and the average effective insert fragment was approximately 60 kb [16]. 

### DNA restriction enzyme digestion and Southern hybridization

The phage DNA extraction, digestion and Southern hybridization were performed according to the methods described by Smeesters and colleagues [17], and Sambrook and Russell, respectively, using the Roche DIG High Prime DNA labeling and detection starter kit I [18].

### Sequencing and analysis

Terminal sequencing of the clones and sub-clone sequencing were performed in Beijing AuGCT Biotechnology Co., Ltd by Sanger sequencing on a 3730XL DNA Analyzer system (Applied Biosystems). Genes and proteins were predicted and annotated by the Prokaryotic Genomes Annotation Pipeline (PGAAP) software packages from NCBI (http://www.ncbi.nlm.nih.gov/genomes/static/Pipeline.html). Plasmid comparison was performed by the Easyfig program [19]. The insert sequence (IS) analysis was performed by IS-FINDER (https://www-is.biotoul.fr/).

### Nucleotide sequence accession number

**Figure 1:**
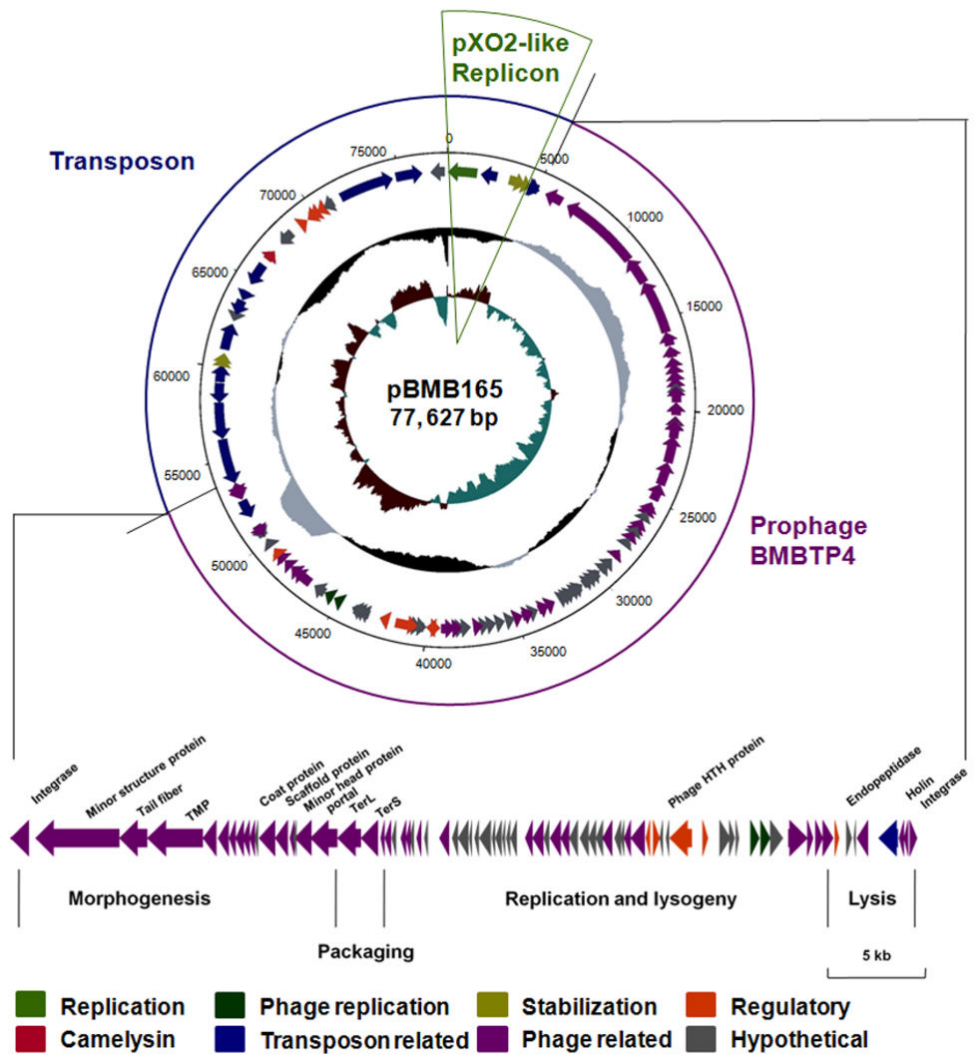
Circular representation of plasmid pBMB165 and graphical representation of the annotation and the structure of the prophage BMBTP3. The inner circle represents the GC bias [(G - C)/(G + C)], with positive and negative values in reddish brown and cobalt blue, respectively; the second circle represents the GC-content, with positive and negative values in grey and black, respectively; and the outer circle represents the predicted genes on the reverse and forward arrows. The pXO2-like replicon is highlighted with a green arching frame. Regions of transposon and prophage BMBTP3 are annotated beside the corresponding arrows and separated by straight lines. The main functional genes of BMBTP3 are annotated above the extended corresponding arrows at the bottom. Different structural and functional regions are annotated and separated by vertical lines. Color coding for the genes is as follows: olive green, plasmid replication; deep green, prophage replication; deep yellow, plasmid stabilization system; orange, regulatory; red, a predicted camelysin; blue, mobile DNA; purple, phage related; grey, hypothetical protein. The outer scale is marked in kilobases.

The full-length 77,627 bp pBMB165 sequence and annotation (Figure 1) has been deposited in the GenBank database under accession number CP002178.

## Results and Discussion

### Complete cloning of pBMB165 with a contig linkage map by BAC libraries

*B. thuringiensis* YBT-1765 has three plasmids, and the largest one is pBMB165 (Table 1, [15]). Previously, we constructed the total plasmid and genomic BAC libraries of YBT-1765, and obtained a 20 kb replication-related fragment [15]. To completely clone the plasmid, we designed a set of PCR primers according to the BAC clones (Table S1), and screened the libraries. After three rounds of screening we obtained 16 BAC clones, including five plasmid BAC clones and 11 genomic BAC clones (Table 1). By comparing the *Bam*HI and/or *Hin*dIII restriction profiles of the inserted fragments, we constructed their linked overlapping relationship, and subsequently created a physical map of pBMB165 (Figure S1). Finally, we selected two clones for DNA sequencing, pBMB165B8 and pBMB165B11, with insert DNA sizes of 69 kb and 35 kb, respectively, which covered the entire pBMB165 plasmid. After sequencing and assembling, the clones pBMB165B8 and pBMB165B11 covered the entire genome of pBMB165 (77,627 bp). 

**Table 1:** Bacterial strains and plasmids used in this study.

Strains	Organism	Plasmids	Insert Fragment Size (kb)	Library Type	Reference
YBT-1765	*B. thuringiensis*	pBMB165, pBMB175 and a uncharacterized plasmid	-	-	[15,31]
EMB165A1	*E. coli*	pBMB165A1	16	Total plasmid	This work
EMB165A2	*E. coli*	pBMB165A2	20	Total plasmid	This work
EMB165A3	*E. coli*	pBMB165A3	42	Total plasmid	This work
EMB165A4	*E. coli*	pBMB165A4	52	Total plasmid	This work
EMB165A5	*E. coli*	pBMB165A5	58	Total plasmid	This work
EMB165B6	*E. coli*	pBMB165B6	58	Total genome	This work
EMB165B7	*E. coli*	pBMB165B7	55	Total genome	This work
EMB165B8	*E. coli*	pBMB165B8	69	Total genome	This work
EMB165B9	*E. coli*	pBMB165B9	25	Total genome	This work
EMB165B10	*E. coli*	pBMB165B10	15	Total genome	This work
EMB165B11	*E. coli*	pBMB165B11	35	Total genome	This work
EMB165B12	*E. coli*	pBMB165B12	27	Total genome	This work
EMB165B13	*E. coli*	pBMB165B13	35	Total genome	This work
EMB165B14	*E. coli*	pBMB165B14	35	Total genome	This work
EMB165B15	*E. coli*	pBMB165B15	25	Total genome	This work
EMB165B16	*E. coli*	pBMB165B16	40	Total genome	This work

As large plasmids always have a low copy number and contain many repeat sequences, we used the BAC clones to construct the linked scaffolds and finally completely cloned the large plasmid pBMB165. The entire sequence of pBMB165 could also be accurately assembled by sequencing the BAC clones. This method of cloning using a whole genome BAC library is not limited to cloning large plasmids, but can also be used to clone other large fragments, such as lysogenic phage [20], the biosynthetic gene cluster of Zwittermicin A [10], and Thuringiensin [9]. Using the selected BAC clones, we validated the function of the biosynthetic gene cluster of Zwittermicin A [10] and Thuringiensin [9].

In this work, we found that the genomic BAC library showed a better coverage than the plasmid library. This is due to that we did not extract a high quality total plasmid DNA of *B. thuringiensis* YBT-1765. As in theory, the plasmid library with a high quality of plasmid DNA must be better than the total genomic DNA library for the plasmid cloning. Even there are some excellent works which described *B. thuringiensis* plasmid extraction [21,22], in our previous work we suffered that extraction of complete and total plasmids from *B. thuringiensis* is a hard work, especially from those *B. thuringiensis* strains with many plasmids and large plasmids (data not shown). Considering the complication of the *B. thuringiensis* plasmid content and for the researchers who cannot prepare a high quality total plasmid DNA, the genomic BAC library would taken as an alternative method for the large plasmid cloning despite a possible lower frequency of screening.

### Sequence analysis revealed pBMB165 consists of abundant mobile genetic elements

Sequence analysis showed that pBMB165 is a circular 77,627 bp plasmid with a GC-content of 35.36%, which is similar to the genome of the *Bc* group. It encodes 103 open reading frames (ORFs), with an average ORF length of 754 bp (Figure 1). There are 71 predicted ORFs that have similarity to proteins with known functions. Our previous work demonstrated that the replicon of pBMB165 consists of a replication initiation protein (Rep165, ORF005), an origin of replication (*ori165*), and a region of iterons, which belongs to the pAMβ1 family with high homology to the pXO2 replicon. ORF015 and ORF020 were shown to be involved in plasmid stability [15]. Interestingly, as well as the replicon, pBMB165 consists of abundant mobile genetic elements, divided into two parts (Figure 1). The first part consists of an approximately 50 kb prophage-related region, named BMBTP3 (76 ORFs, ORF035-410), and the second part is a nearly 23 kb mobile region that gathers around the replication region (27 ORFs, ORF005-030, and ORF430-540). 

The BMBTP3 region has 47 ORFs that are predicted to be phage-related proteins (Table S2). 16 ORFs encode structure-related proteins, and thus form a “morphogenesis” module (Figure 1). This module includes the minor structural protein (ORF040), tail fiber protein (ORF045), tail tape measure protein (ORF050), structural protein (ORF060), head-tail adaptor (ORF075), scaffold protein (ORF095), minor head protein (ORF0110) and portal protein (ORF115). The “DNA packaging” module consists of the large and small terminase subunits (ORF120-125); and the “replication and lysogeny” module consists of 49 ORFs with functions in replication, transcription, regulation and recombination. The “lysis” module encodes three proteins involved in cell lysis (ORFs 390-405), two integrases (ORF035 and ORF410) and an insertion sequence element IS5 (ORF395). This shows that the BMBTP3 region contains all the integral components of a phage, and implies that it might be functional (Figure 1).

In the replicon and transposon region, 16 ORFs belonged to the transposon family, including 5 IS elements (Table S3) and a cluster of four ORFs (ORFs 425-440), which have 99% identity with the class II transposable element Tn5401, in addition to two 53 bp terminally inverted repeats (Table S2: the four ORFs correspond to *tnpA*, *tnpI*, *orf1*, and *orf2*, respectively [23]). This region also includes some transcriptional regulators and DNA/RNA binding proteins, and a putative pathogenic factor camelysin (ORF470, Table S2), which may enhance the toxicity of the Cyt proteins [24,25]. The existence of camelysin indicates that pBMB165 may play an important role in the toxicity of *B. thuringiensis* YBT-1765. 

### Comparative analysis reveals that pBMB165 is a special pXO2-like plasmid

**Figure 2:**
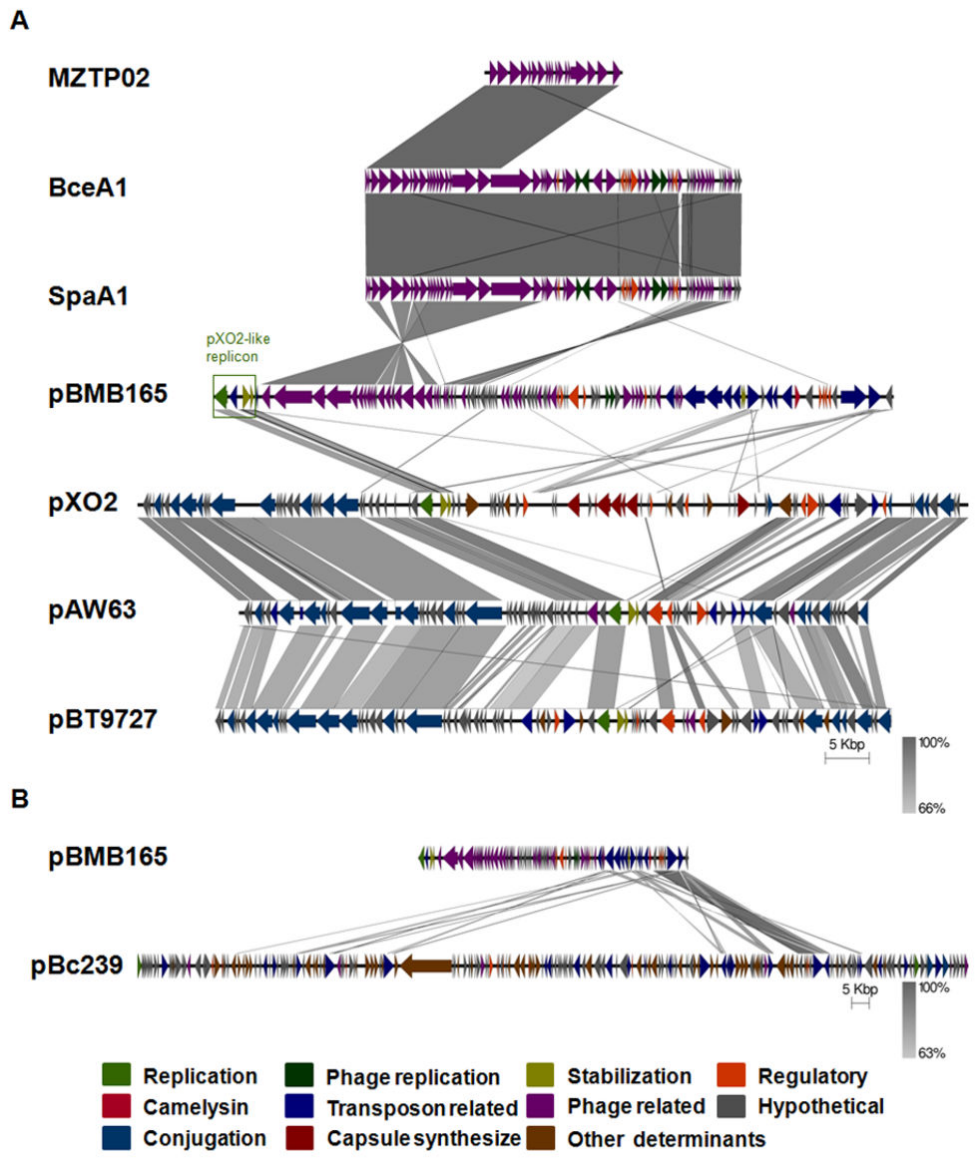
Comparison of pBMB165 and homologous plasmids and phages by Easyfig alignment. Coding Sequences (CDSs) are represented by colored arrows. Predicted functions/homologies are indicated by the color key featured below. The pXO2-like replicon is highlighted with a green frame. Color coding for the genes is as follows: olive green, plasmid replication; deep green, prophage replication; deep yellow, plasmid stabilization system; orange, regulatory; red, a predicted camelysin; blue, mobile DNA; purple, phage related; grey, hypothetical protein; midnight blue, conjugation-related proteins; wine, capsule synthesis related proteins; and brown, other determinants. Highly conserved segments of the plasmids and phages are paired by shaded regions, with the darker shading reflecting a greater amino acid identity, from 66% (A) or 63% (B) to 100%. The regions outside the shaded regions lack homology between plasmids and phages. The outer scale is marked in kilobases.

As mentioned above, the replicon of pBMB165 has homology with the plasmids pXO2, pAW63 and pBT9727 [15], but the ORF annotation shows that there is a high density of transposon genes around the replicon of pBMB165, making it significantly different to pXO2-like plasmids. Whole plasmid comparative analysis shows that in the public databases, pBMB165 has a homologous transposon region to the large *Bc* plasmid pBc239 (Figure 2B), and the BMBTP3 region is almost identical to three reported homogenous *Siphoviridae* family bacteriophages, SpaA1, BceA1 and MZTP02 (Figure 2A). As the phages SpaA1 and BceA1 all have a mosaic genome within another phage MZTP02 [26], the ORF annotation and the comparison both show that BMBTP3 has the same structure as these phages in the morphogenesis and DNA packaging modules, but a totally different structure in the replication, lysogeny and lysis modules (Figures 1 and 2A). This implies that the large plasmid pBMB165 is not an ordinary pXO2-like plasmid, but has a novel organization consisting of a pXO2-like family replicon, a prophage BMBTP3 region, and a series of widespread transposons carrying some functional genes.

This indicates that the commonly descended plasmids may have adopted exogenetic DNA during their long evolutionary history. Alternatively, the plasmids may actively influence the parasitic hosts to maintain a better existence. Although they share the same replication origin, pXO2 carries pathogenic factors [2], and pAW63 and pBT9727 are both conjugative [27,28]; however, these phenotypes are directly or indirectly necessary to ensure the widespread of the plasmids throughout the population. In the case of plasmid pBMB165, it remains unclear how the transposable elements and prophage help to stabilize the plasmid during the evolutionary process, but these different kinds of mobile elements may be beneficial for efficient horizontal gene transfer in the host genome. 

### The inducible prophage integrated in pBMB165 reveals a novel plasmid organization pattern

**Figure 3:**
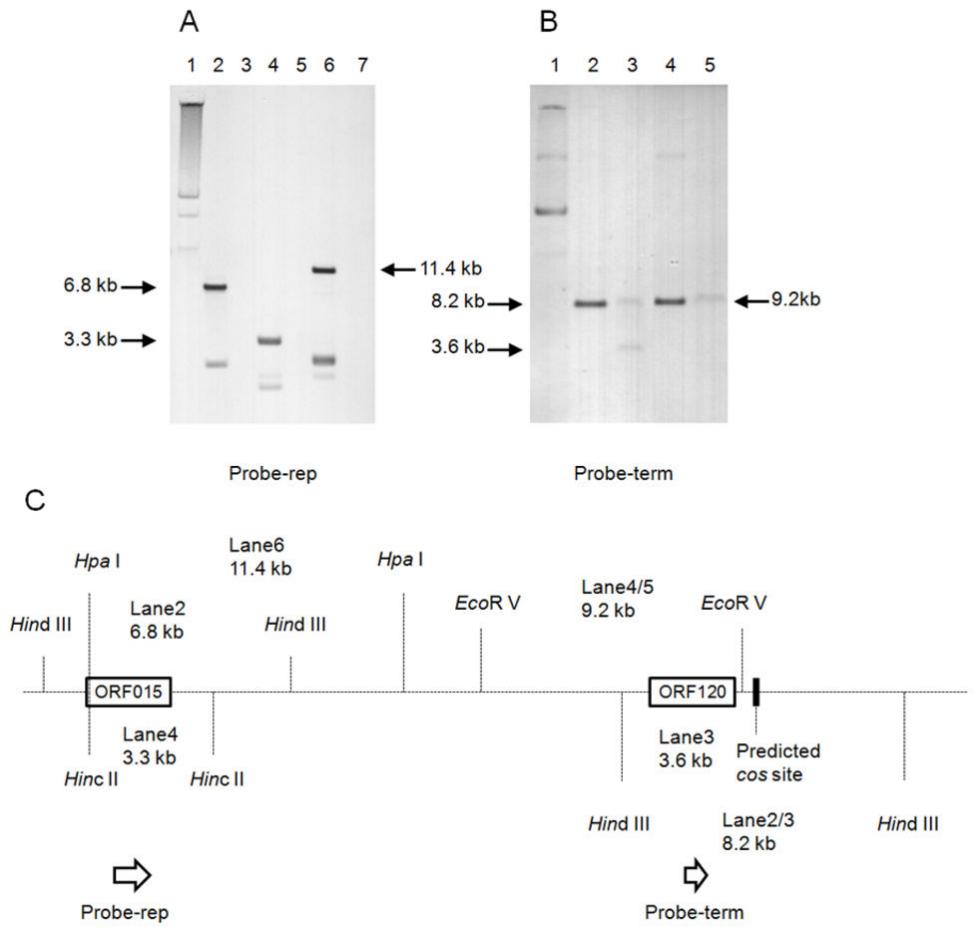
Identifying BMBTP3 among the total induced phage DNA from B. ***thuringiensis* strain YBT-1765**. **A**. Southern hybridization with a replication-associated protein gene specific probe (probe-rep). Lane 1, the total plasmid DNA extracted from YBT-1765; Lane 2, digested total plasmid DNA by *Hin*dIII; Lane 3, digested total induced phage DNA by *Hin*dIII; Lane 4, digested total plasmid DNA by *Hin*cII; Lane 5, digested total induced phage DNA by *Hin*cII; Lane 6, digested total plasmid DNA by *Hpa*I; Lane 7, digested total induced phage DNA by *Hpa*I. **B**. Southern hybridization with a phage terminase large subunit gene specific probe (probe-term). Lane 1, the total plasmid DNA extracted from YBT-1765; Lane 2, digested total plasmid DNA by *Hin*dIII; Lane 3, digested total induced phage DNA by *Hin*dIII; Lane 4, digested total plasmid DNA by *Eco*RV; Lane 5, digested total induced phage DNA by *Eco*RV. The sizes of the signal bands are labeled with arrows. In each lane for total plasmids and digested products we loaded 0.7 μg plasmid DNA (lanes 1, 2, 4, 6 in Figures 3A and 1, 2, 4 in Figure 3B), and for the purified phage DNA and digested products, we loaded 1.3 μg in each lane (lanes 3, 5, 7 in Fig. 3A and 3, 5 in Fig. 3B). **C**. The schematic drawing shows the structure of the restriction fragments with the ORF015 (probe-rep), ORF120 (probe-term) and the predicted cos site. The dashed line denotes the DNA of pBMB165, and the sizes of fragment digested by the restriction enzymes and the predicted cos site.

To discover whether the integrated prophage BMBTP3 is functional or not, we used mitomycin C to induce the phage from *B. thuringiensis* YBT-1765, and subsequently extracted the genomic DNA of the induced phage. Upon performing pulsed field gel electrophoresis, we found that there are at least two components to the DNA at about 40 kb (data not shown). To determine whether the pBMB165 prophage is inducible, we performed Southern hybridization. The total plasmid DNA and the induced phage genomic DNA from YBT-1765 were used as templates, after digestion by *Hin*dIII, *Hin*cII, *Hpa*I and *Eco*RV. Two specific probes were designed based on a plasmid replication-associated protein gene (ORF015, probe-rep, located on 3806-5420,) and a phage terminase gene (ORF120, probe-term, located on 23551-24099, Figure 3C). The predicted sizes of the restriction fragments containing the probes are shown as a schematic in Figure 3C. As BMBTP3 has a very similar DNA packaging module to SpaA1, we found a 9 bp region (nucleotides 25704-25712, 5’-TGGAGGAGG -3’) adjacent to the DNA packaging module that has up to 100% homology with the single-stranded cohesive (*cos*) ends of phage SpaA1 [26], which has been annotated “predicted *cos* site”.

When using probe-rep, all the plasmid-template lanes showed hybridized positive signal bands, with the main bands at 6.8 kb, 3.3 kb and 11.4 kb as predicted by the sequence restriction site analysis, while the phage-template lanes did not (Figure 3A). Using the probe-term, both template lanes have positive bands, and lane 3 showed two positive bands (Figure 3B). It is thought that the linear phage DNA with *cos* end sites would form a circular molecule. As the predicted *cos* site of BMBTP3 was inside the *Hin*dIII digested fragment and flanked the probe-term, the two signal bands on the Southern blot could result from the linear-induced BMBTP3 DNA, giving a band size of 3.6 kb, and the circular-induced BMBTP3 DNA resulting from adhesion with the *cos* site, giving a band size of 8.2 kb (Figure 3C). In other words, the 3.6 kb signal band caused by the “*cos*” site confirmed that the positive signals were due to the DNA of the induced phage BMBTP3, and not contamination by plasmid DNA. This result shows that BMBTP3 is an inducible prophage, and uses the same site-specific mechanism as SpaA1 for packaging.

Plasmids and bacteriophage can contribute important biological properties to their bacterial hosts, and therefore are the motive force for horizontal gene transfer among bacteria. However, there is not a direct intersection between them: almost all of the reported bacteriophage usually integrate into the host chromosome, and only a few have been reported that do not integrate, but exist as circular or linear plasmids called “phagemids” [17,29]. While some plasmids have been shown to carry some integrase homology and other phage-related genes, these genes are involved with the replication and transmission of the plasmid, and not integration of the prophage [30]. After conducting analysis using public databases, we did not find any other plasmid from the *Bacillus* sp. that carries a complete prophage-integrated region. 

Here, we describe a novel pattern of plasmid organization, where the characteristic pXO2-like plasmid pBMB165 is integrated by an inducible prophage BMBTP3, and the rest of the plasmid is abundant in transposable elements. As this novel plasmid organization consists of three different kinds of mobile element, including the plasmid replicon, prophage and transposable elements (Figure 2A and 2B), it suggests that the large plasmid pBMB165 may be an intermediate product of recombination events: after the phage BMBTP3 infected the *Bt* host YBT-1765 and entered the lysogenic state, it degenerated or was inhibited during the co-evolutionary process with the host and did not develop into a free self-replicated phagemid, while the recombination event that occurred between the pXO2-like replicon and some transposons offered appropriate sites for the integration of the degenerated phage BMBTP3. As the pXO2-like replicon is functional, the final recombination among the three mobile elements benefited the phage BMBTP3 and allowed it to recover genetic stability as a temperate prophage via the large plasmid pBMB165. 

Compared with other induced phages of YBT-1765, the lower abundance of the inducible prophage BMBTP3 also suggests that the novel recombined composition of pBMB165 has a young evolutionary history, and could evolve further, either to lose the function of the pXO2-like replicon and be a real “phagemid” or to be a pure plasmid integrated into a degenerated prophage. 

In conclusion, we cloned a large native plasmid pBMB165 with a novel organization pattern, which reveals that some prophages may use the plasmid replicon to maintain genetic stability. Our findings provide new information that updates our understanding of co-evolution between bacterial plasmids and bacteriophage.

## Supporting Information

Figure S1 **The circular physical contig linkage map of plasmid pBMB165.** The area denoted with an arrow is the replication region of plasmid pBMB165. The inner, circular double line is the plasmid pBMB165, and restriction enzyme sites *Hin*dIII and *Bam*HI are indicated with dotted lines. The dashed line arcs, which are denoted (1) to (5), are the pBMB165A1-A5 clones (in numerical order) from the total plasmid BAC library. The long dashed line arcs, which are denoted (6) to (16), are the pBMB165B6-B16 clones (in numerical order) from the genomic BAC library. The solid line arcs, denoted (8) and (11), are clones pBMB165B8 and pBMB165B11.
(TIF)

Table S1 **Primers used in this study.** The table contains the oligonucleotide sequence of all the primers used for the BAC libraries screening as described in the main text. The primers were designed using the Primer Premier 5.0 software (http://www.premierbiosoft.com/primerdesign/index.html). The suffix “-1” means the forward primer, “-2” means the reverse primer.
(XLSX)

Table S2 **Predicted genes in pBMB165.** The table contains the list of predicted genes in the large plasmid pBMB165. The annotation result shows two major regions of apparent different origins, the transposon and replicon region, and the prophage BMBTP4 region. It reveals a novel plasmid organization.
(XLS)

Table S3 **Details of IS elements on plasmid pBMB165.** Included in this table are the brief descriptions of the IS elements on transposon and replicon region in the plasmid pBMB165 described in the main text.
(XLS)

## References

[1] Rasko DA, Altherr MR, Han CS, Ravel J (2005) Genomics of the Bacillus cereus group of organisms. FEMS Microbiol Rev 29: 303-329. doi:10.1016/j.fmrre.2004.12.005. PubMed: 15808746. 15808746

[2] Koehler TM (2002) Bacillus anthracis genetics and virulence gene regulation. Curr Top Microbiol Immunol 271: 143-164. doi:10.1007/978-3-662-05767-4_7. PubMed: 12224521. 12224521

[3] Stenfors Arnesen LP, Fagerlund A, Granum PE (2008) From soil to gut: Bacillus cereus and its food poisoning toxins. FEMS Microbiol Rev 32: 579-606. doi:10.1111/j.1574-6976.2008.00112.x. PubMed: 18422617. 18422617

[4] Tanimoto K, Ike Y (2008) Complete nucleotide sequencing and analysis of the 65-kb highly conjugative Enterococcus faecium plasmid pMG1: identification of the transfer-related region and the minimum region required for replication. FEMS Microbiol Lett 288: 186-195. doi:10.1111/j.1574-6968.2008.01342.x. PubMed: 18795955. 18795955

[5] Schnepf E, Crickmore N, Van Rie J, Lereclus D, Baum J et al. (1998) Bacillus thuringiensis and its pesticidal crystal proteins. Microbiol Mol Biol Rev 62: 775-806. PubMed: 9729609. 9729609 10.1128/mmbr.62.3.775-806.1998 PMC98934

[6] Guo S, Liu M, Peng D, Ji S, Wang P et al. (2008) New strategy for isolating novel nematicidal crystal protein genes from Bacillus thuringiensis strain YBT-1518. Appl Environ Microbiol 74: 6997-7001. doi:10.1128/AEM.01346-08. PubMed: 18820056. 18820056 PMC2583473

[7] Loeza-Lara PD, Benintende G, Cozzi J, Ochoa-Zarzosa A, Baizabal-Aguirre VM et al. (2005) The plasmid pBMBt1 from Bacillus thuringiensis subsp. darmstadiensis (INTA Mo14-4) replicates by the rolling-circle mechanism and encodes a novel insecticidal crystal protein-like gene. Plasmid 54: 229-240. doi:10.1016/j.plasmid.2005.04.003. PubMed: 15970328. 15970328

[8] Zhao C, Luo Y, Song C, Liu Z, Chen S et al. (2007) Identification of three Zwittermicin A biosynthesis-related genes from Bacillus thuringiensis subsp. kurstaki strain YBT-1520. Arch Microbiol 187: 313-319. doi:10.1007/s00203-006-0196-3. PubMed: 17225146. 17225146

[9] Liu XY, Ruan LF, Hu ZF, Peng DH, Cao SY et al. (2010) Genome-wide screening reveals the genetic determinants of an antibiotic insecticide in Bacillus thuringiensis. J Biol Chem 285: 39191-39200. doi:10.1074/jbc.M110.148387. PubMed: 20864531. 20864531 PMC2998090

[10] Luo Y, Ruan LF, Zhao CM, Wang CX, Peng DH et al. (2011) Validation of the intact zwittermicin A biosynthetic gene cluster and discovery of a complementary resistance mechanism in Bacillus thuringiensis. Antimicrob Agents Chemother 55: 4161-4169. doi:10.1128/AAC.00111-11. PubMed: 21730118. 21730118 PMC3165285

[11] Zhong C, Peng D, Ye W, Chai L, Qi J et al. (2011) Determination of plasmid copy number reveals the total plasmid DNA amount is greater than the chromosomal DNA amount in Bacillus thuringiensis YBT-1520. PLOS ONE 6: e16025. doi:10.1371/journal.pone.0016025. PubMed: 21283584. 21283584 PMC3026805

[12] Berry C, O'Neil S, Ben-Dov E, Jones AF, Murphy L et al. (2002) Complete sequence and organization of pBtoxis, the toxin-coding plasmid of Bacillus thuringiensis subsp. israelensis. Appl Environ Microbiol 68: 5082-5095. doi:10.1128/AEM.68.10.5082-5095.2002. PubMed: 12324359. 12324359 PMC126441

[13] Van der Auwera GA, Andrup L, Mahillon J (2005) Conjugative plasmid pAW63 brings new insights into the genesis of the Bacillus anthracis virulence plasmid pXO2 and of the Bacillus thuringiensis plasmid pBT9727. BMC Genomics 6: 103. doi:10.1186/1471-2164-6-103. PubMed: 16042811. 16042811 PMC1196294

[14] Hu X, Van der Auwera G, Timmery S, Zhu L, Mahillon J (2009) Distribution, diversity, and potential mobility of extrachromosomal elements related to the Bacillus anthracis pXO1 and pXO2 virulence plasmids. Appl Environ Microbiol 75: 3016-3028. doi:10.1128/AEM.02709-08. PubMed: 19304837. 19304837 PMC2681636

[15] Huang J, Guo S, Mahillon J, Van der Auwera GA, Wang L et al. (2006) Molecular characterization of a DNA fragment harboring the replicon of pBMB165 from Bacillus thuringiensis subsp. tenebrionis. BMC Genomics 7: 270. doi:10.1186/1471-2164-7-270. PubMed: 17059605. 17059605 PMC1626470

[16] Wang L, Guo S, Huang J, Yu Z, Sun M (2008) Cloning and physical map construction of a large plasmid pBMB165 in Bacillus thuringiensis. Wei Sheng Wu Xue Bao 48: 15-20. PubMed: 18338570. 18338570

[17] Smeesters PR, Drèze PA, Bousbata S, Parikka KJ, Timmery S et al. (2011) Characterization of a novel temperate phage originating from a cereulide-producing Bacillus cereus strain. Res Microbiol 162: 446-459. doi:10.1016/j.resmic.2011.02.009. PubMed: 21349326. 21349326

[18] Sambrook J, Russell DW, editors (2001) Molecular cloning: a laboratory manual, 3rd ed. Cold Spring Harbor, NY: Cold Spring Harbor Laboratory Press.

[19] Sullivan MJ, Petty NK, Beatson SA (2011) Easyfig: a genome comparison visualizer. Bioinformatics 27: 1009-1010. doi:10.1093/bioinformatics/btr039. PubMed: 21278367. 21278367 PMC3065679

[20] Chikerema SM, Pfukenyi DM, Hang'ombe BM, L'Abee-Lund TM, Matope G (2012) Isolation of Bacillus anthracis from soil in selected high-risk areas of Zimbabwe. J Appl Microbiol 113: 1389-1395. doi:10.1111/jam.12006. PubMed: 22984812. 22984812

[21] Andrup L, Barfod KK, Jensen GB, Smidt L (2008) Detection of large plasmids from the Bacillus cereus group. Plasmid 59: 139-143. doi:10.1016/j.plasmid.2007.11.005. PubMed: 18179822. 18179822

[22] Reyes-Ramírez A, Ibarra JE (2008) Plasmid patterns of Bacillus thuringiensis type strains. Appl Environ Microbiol 74: 125-129. doi:10.1128/AEM.02133-07. PubMed: 18024687. 18024687 PMC2223206

[23] Baum JA (1994) Tn5401, a new class II transposable element from Bacillus thuringiensis. J Bacteriol 176: 2835-2845. PubMed: 7514590. 7514590 10.1128/jb.176.10.2835-2845.1994 PMC205437

[24] Nisnevitch M, Cohen S, Ben-Dov E, Zaritsky A, Sofer Y et al. (2006) Cyt2Ba of Bacillus thuringiensis israelensis: activation by putative endogenous protease. Biochem Biophys Res Commun 344: 99-105. doi:10.1016/j.bbrc.2006.03.134. PubMed: 16630537. 16630537

[25] Nisnevitch M, Sigawi S, Cahan R, Nitzan Y (2010) Isolation, characterization and biological role of camelysin from Bacillus thuringiensis subsp. israelensis. Curr Microbiol 61: 176-183. doi:10.1007/s00284-010-9593-6. PubMed: 20127334. 20127334

[26] Swanson MM, Reavy B, Makarova KS, Cock PJ, Hopkins DW et al. (2012) Novel bacteriophages containing a genome of another bacteriophage within their genomes. PLOS ONE 7: e40683. doi:10.1371/journal.pone.0040683. PubMed: 22815791. 22815791 PMC3398947

[27] Van der Auwera GA, Timmery S, Mahillon J (2008) Self-transfer and mobilisation capabilities of the pXO2-like plasmid pBT9727 from Bacillus thuringiensis subsp. konkukian 97-27. Plasmid 59: 134-138. doi:10.1016/j.plasmid.2007.11.007. PubMed: 18272219. 18272219

[28] Wilcks A, Jayaswal N, Lereclus D, Andrup L (1998) Characterization of plasmid pAW63, a second self-transmissible plasmid in Bacillus thuringiensis subsp. kurstaki HD73. Microbiology 144 ( 5): 1263-1270. doi:10.1099/00221287-144-5-1263. PubMed: 9611801. 9611801

[29] Casjens S (2003) Prophages and bacterial genomics: what have we learned so far? Mol Microbiol 49: 277-300. doi:10.1046/j.1365-2958.2003.03580.x. PubMed: 12886937. 12886937

[30] Boltner D, MacMahon C, Pembroke JT, Strike P, Osborn AM (2002) R391: a conjugative integrating mosaic comprised of phage, plasmid, and transposon elements. J Bacteriol 184: 5158-5169 10.1128/JB.184.18.5158-5169.2002 PMC135318 12193633

[31] Huang J, Han D, Yu Z, Sun M (2007) A novel cryptic plasmid pBMB175 from Bacillus thuringiensis subsp. tenebrionis YBT-1765. Arch Microbiol 188: 47-53. doi:10.1007/s00203-007-0222-0. PubMed: 17310366. 17310366

