# Supplementary figures and images for: Cloning and Analysis of a Large Plasmid pBMB165 from Bacillus thuringiensis Revealed a Novel Plasmid Organization
Source: PLoS One. 2013 Dec 2;8(12):e81746. doi: 10.1371/journal.pone.0081746 (PMC3847046; doi:10.1371/journal.pone.0081746)

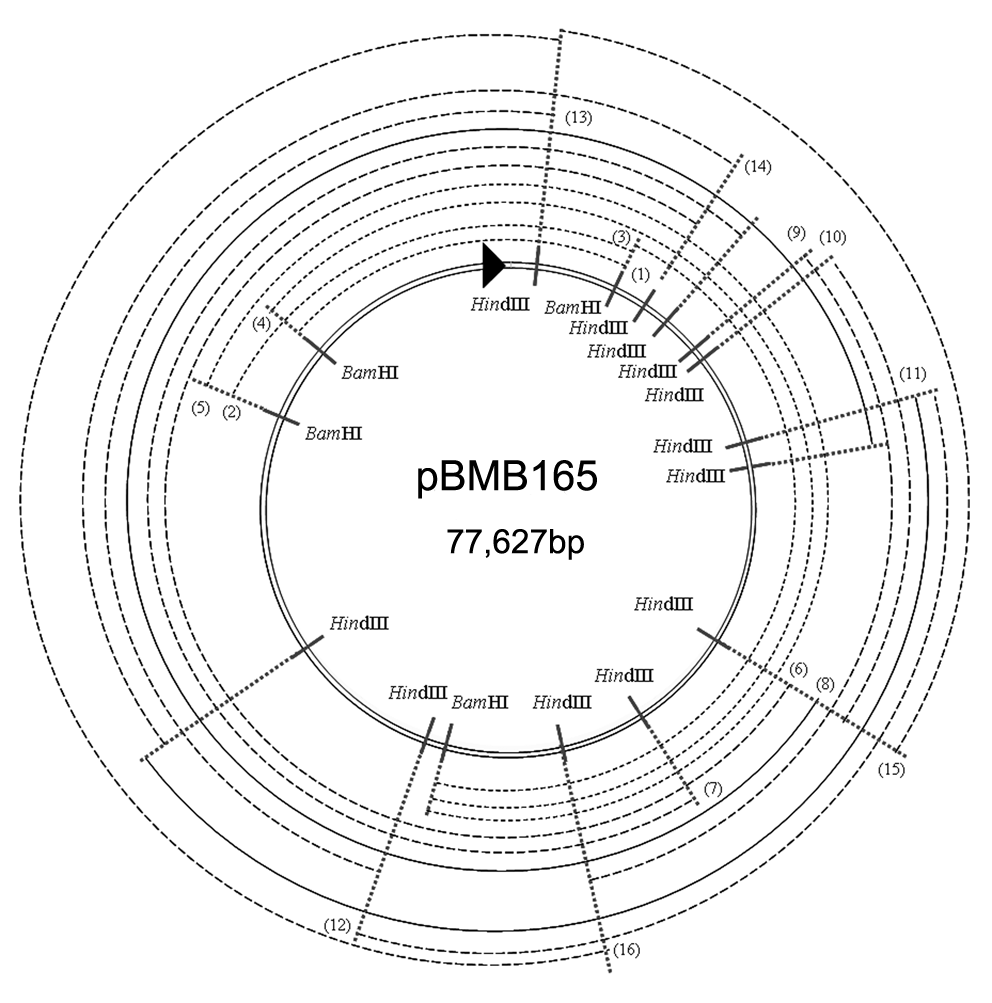

Supplement: Figure S1 — The circular physical contig linkage map of plasmid pBMB165. The area denoted with an arrow is the replication region of plasmid pBMB165. The inner, circular double line is the plasmid pBMB165, and restriction enzyme sites HindIII and BamHI are indicated with dotted lines. The dashed line arcs, which are denoted (1) to (5), are the pBMB165A1-A5 clones (in numerical order) from the total plasmid BAC library. The long dashed line arcs, which are denoted (6) to (16), are the pBMB165B6-B16 clones (in numerical order) from the genomic BAC library. The solid line arcs, denoted (8) and (11), are clones pBMB165B8 and pBMB165B11. (TIF) [file pone.0081746.s001.tif]
